# High Specificity Wearable Device With Photoplethysmography and Six-Lead Electrocardiography for Atrial Fibrillation Detection Challenged by Frequent Premature Contractions: DoubleCheck-AF

**DOI:** 10.3389/fcvm.2022.869730

**Published:** 2022-04-06

**Authors:** Justinas Bacevicius, Zygimantas Abramikas, Ernestas Dvinelis, Deimile Audzijoniene, Marija Petrylaite, Julija Marinskiene, Justina Staigyte, Albinas Karuzas, Vytautas Juknevicius, Rusne Jakaite, Viktorija Basyte-Bacevice, Neringa Bileisiene, Andrius Solosenko, Daivaras Sokas, Andrius Petrenas, Monika Butkuviene, Birute Paliakaite, Saulius Daukantas, Andrius Rapalis, Germanas Marinskis, Eugenijus Jasiunas, Angeliki Darma, Vaidotas Marozas, Audrius Aidietis

**Affiliations:** ^1^Institute of Clinical Medicine, Faculty of Medicine, Vilnius University, Vilnius, Lithuania; ^2^Center of Cardiology and Angiology, Vilnius University Hospital Santaros Klinikos, Vilnius, Lithuania; ^3^Biomedical Engineering Institute, Kaunas University of Technology, Kaunas, Lithuania; ^4^Center of Informatics and Development, Vilnius University Hospital Santaros Klinikos, Vilnius, Lithuania; ^5^Heart Center Leipzig at University of Leipzig and Leipzig Heart Institute, Leipzig, Germany; ^6^Department of Electronics Engineering, Kaunas University of Technology, Kaunas, Lithuania

**Keywords:** wrist-worn device, multiple-lead portable ECG, telemedicine, mhealth, remote monitoring, digital health

## Abstract

**Background:**

Consumer smartwatches have gained attention as mobile health (mHealth) tools able to detect atrial fibrillation (AF) using photoplethysmography (PPG) or a short strip of electrocardiogram (ECG). PPG has limited accuracy due to the movement artifacts, whereas ECG cannot be used continuously, is usually displayed as a single-lead signal and is limited in asymptomatic cases.

**Objective:**

DoubleCheck-AF is a validation study of a wrist-worn device dedicated to providing both continuous PPG-based rhythm monitoring and instant 6-lead ECG with no wires. We evaluated its ability to differentiate between AF and sinus rhythm (SR) with particular emphasis on the challenge of frequent premature beats.

**Methods and Results:**

We performed a prospective, non-randomized study of 344 participants including 121 patients in AF. To challenge the specificity of the device two control groups were selected: 95 patients in stable SR and 128 patients in SR with frequent premature ventricular or atrial contractions (PVCs/PACs). All ECG tracings were labeled by two independent diagnosis-blinded cardiologists as “AF,” “SR” or “Cannot be concluded.” In case of disagreement, a third cardiologist was consulted. A simultaneously recorded ECG of Holter monitor served as a reference. It revealed a high burden of ectopy in the corresponding control group: 6.2 PVCs/PACs per minute, bigeminy/trigeminy episodes in 24.2% (31/128) and runs of ≥3 beats in 9.4% (12/128) of patients. AF detection with PPG-based algorithm, ECG of the wearable and combination of both yielded sensitivity and specificity of 94.2 and 96.9%; 99.2 and 99.1%; 94.2 and 99.6%, respectively. All seven false-positive PPG-based cases were from the frequent PVCs/PACs group compared to none from the stable SR group (*P* < 0.001). In the majority of these cases (6/7) cardiologists were able to correct the diagnosis to SR with the help of the ECG of the device (*P* = 0.012).

**Conclusions:**

This is the first wearable combining PPG-based AF detection algorithm for screening of AF together with an instant 6-lead ECG with no wires for manual rhythm confirmation. The system maintained high specificity despite a remarkable amount of frequent single or multiple premature contractions.

## Introduction

Atrial fibrillation (AF) is closely associated with an ageing population and its prevalence is expected to double by 2060 to 17.9 million in the European Union alone (1). Consequently, the burden of thromboembolic events, heart failure, bleeding and other major implications may arise. Health care resources must adapt to large-scale early diagnosis and individualized state-of-the-art treatment. To comply with it, the European Society of Cardiology upgraded the recommendation class for systematic electrocardiography (ECG) screening to detect AF in individuals aged ≥75 years, or those at high risk of stroke from 'may be considered' (class IIb) to 'should be considered' (class IIa) (2). Opportunistic screening for AF by pulse taking or ECG rhythm strip in patients ≥65 years of age remains 'recommended' (class I). The problem of insufficient examination for AF is also relevant to secondary prevention. As revealed by an international survey, in 40% of European countries only conventional ECG without long-term cardiac monitoring is the most common method to exclude AF after transient ischemic attack (3).

Accelerated by the Covid-19 pandemic (4) new wearable technologies have the potential to expand the availability of medical care (5, 6), reduce health inequities in remote areas (7) and integrate into the workflow of dedicated AF teams (8). However, clinicians need to assess the cost-effectiveness, regulatory approval, specific clinical applications, patient expectations, data logistics and other issues of each mobile health (mHealth) tool (9). As implied by the World Health Organization, the cost of finding a case (including the diagnosis and the treatment of the diagnosed) should be economically balanced in relation to possible expenditure on medical care as a whole (10). We are convinced that major real-world flaws of wearables are driven by false-positive cases, which require numerous additional resources.

In the DoubleCheck-AF study we present a wrist-worn device dedicated to providing both extensive photoplethysmography-based (PPG) rhythm monitoring and reliable decision establishment with a wearable 6-lead limb-like ECG. The aim of this paper was to evaluate whether the described system has an acceptable ability to differentiate between AF and sinus rhythm (SR) when challenged by a substantial group of patients with premature ventricular or atrial contractions (PVCs/PACs), that are often underestimated in clinical trials.

## Materials and Methods

### Study Design and Recruitment

DoubleCheck-AF is a single-center, non-randomized validation study with a prospective case-control model. It was carried out in accordance with the Declaration of Helsinki. A regional bioethics committee approved the study with registration No. 158200-18/7-1052-557. All enrolled patients provided a written informed consent. The study is registered at ClinicalTrials.gov (NCT04281927).

Patients were recruited from inpatient and outpatient wards of Cardiology Department at Vilnius University Hospital Santaros Klinikos. Inclusion criteria were adult patients (18 to 99 years) with a current ECG-based diagnosis of AF, sinus rhythm (SR) or SR with frequent PVCs/PACs (at least one ectopic beat in 2 min). Subjects with a regular pulse wave despite AF (e.g., paced ventricular beats) or with other arrhythmia as well as those who refused to sign or could not give an informed consent were excluded.

### Measurements

**Figure 1:**
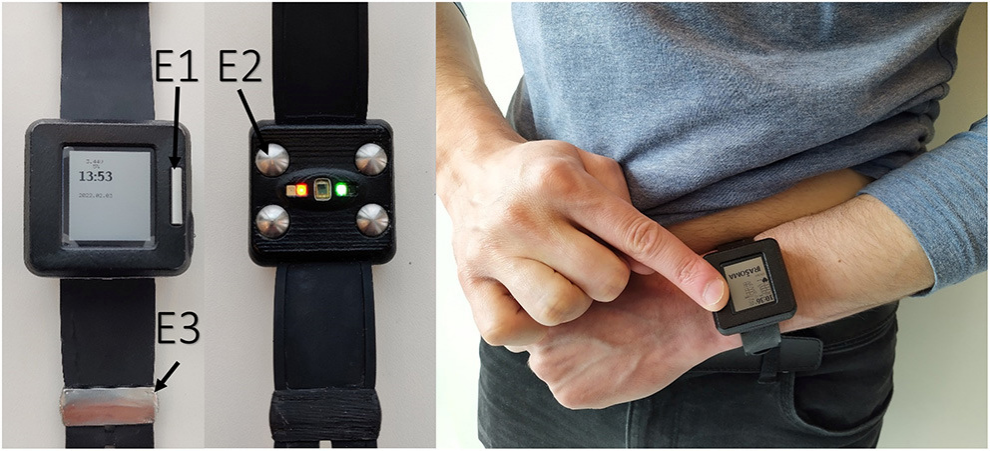
Prototype of the wearable device (left panel); acquiring of 6-lead ECG without any wires (right panel).

The wrist-worn device integrates two types of sensors: PPG for continuous screening of AF and on-demand 6-lead ECG with no wires for rhythm confirmation (Figure 1).

The automatic PPG-based algorithm indicates whether AF is suspected as described previously (11, 12). An embedded PPG sensor uses a green light-emitting diode and a photodetector to continuously measure changes in blood flow. The algorithm relies on the analysis of peak-to-peak intervals, extracted using an adaptive threshold for peak detection. The AF detector has several solutions to reduce the false alarm rate, including filtering of ectopic beats, bigeminy suppression, sinus arrhythmia suppression and continuous signal quality assessment. The latter analyses each detected PPG pulse, identifies artifacts and enables reliable long-term monitoring for AF. The algorithm for AF detection is flexible with respect to the briefest duration of possible AF episode (12). In this study, the algorithm was tuned to detect as short AF episodes as 30 s, accounting to the clinical definition of the minimal duration of paroxysmal AF. Therefore, the PPG-based algorithm triggers the AF alarm after an average duration of 30 s in AF if the condition of sufficient signal quality index is met (11). It should be noted that the duration from the onset of AF to the alarm may vary depending on the heart rate irregularity. That is, the alarm can be triggered as soon as after 5 s in case of highly irregular AF, but no later than after 1 min in case of AF with very low irregularity of heartbeats. Once the PPG-based algorithm detects a possible AF episode, it triggers a vibration alarm for the user.

**Figure 2:**
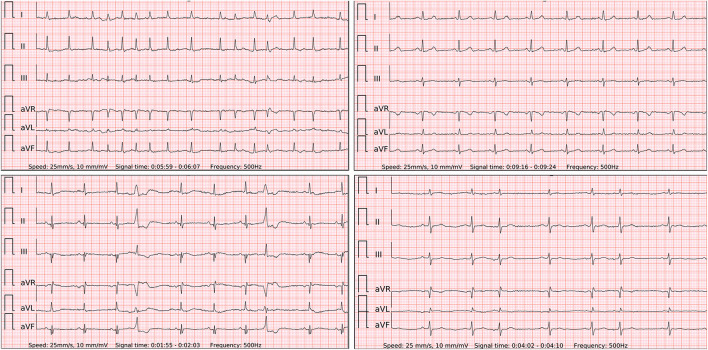
The 6-lead ECGs recorded by the wearable device with the examples of atrial fibrillation (top left panel); stable sinus rhythm (SR) (top right panel); SR with frequent premature ventricular contractions (lower left panel); SR with frequent premature atrial contractions (lower right panel).

Following the alarm notification, a wearable 6-lead ECG is acquired (Figure 2). During our study, even if no notification occurred in at least 1 min of wearing it, an ECG was recorded as well to confirm the rhythm and investigate the method.

The device has three electrodes: two on the outer surface and one on the inner surface next to the PPG sensor (Figure 1). The wearable ECG was recorded by touching one electrode on the upper surface with the right index finger and holding another electrode on the left upper abdomen under the rib cage (Figure 1). In this way, the Einthoven leads I and II were measured. The lead III was calculated according to Kirchhoff's law. Goldberger augmented limb leads aVR, aVL and aVF were also calculated from the measured leads. ECG strips were registered at the intervals of contact between the electrodes and the patient's body. The sudden drop in bioimpedance recorded in a separate channel helped to detect ECG recording events in the continuous multichannel signal. All signals were recorded in a file within the internal memory of the device using a secure GDF (General Data Format) (13). Manual ECG rhythm assessment was performed using dedicated software based on the presence or absence of P-waves and the regularity of QRS complexes.

Diagnostic measures of both PPG-based and wearable ECG-based AF detection methods were calculated separately. Alternatively, the methods were also evaluated together as a “double-check” system for detection of AF. In the latter strategy an embedded PPG-based detector ensures continuous monitoring for AF episodes. If it suspects AF and alarms the patient, only then a matched wearable 6-lead ECG is included in further data analysis for diagnosis confirmation. Such approach does not correct false-negative cases of the PPG algorithm, i.e., sensitivity, but may add great value in reducing the number of false-positive cases of the PPG algorithm, i.e., improving specificity.

All participants used the wearable to record at least a total of 2 min of PPG and 2 min of 6-lead standard-limb-like ECG. In addition, each subject was simultaneously monitored with a validated ECG Holter monitor (eMotion Faros, Kuopio, Finland), which recorded a continuous 3-lead ECG. The ECG of Holter monitor served as a gold standard test for cardiologists to verify the heart rhythm and provide a comparable reference to the PPG-based algorithm and wearable 6-lead ECG of the studied device.

### Data Analysis

Two independent diagnosis-blinded cardiologists labeled all ECG tracings as “AF,” “SR” or “Cannot be concluded.” In case of disagreement, a third diagnosis-blinded cardiologist was consulted. The ECG tracings of the studied device and the gold standard Holter have a different number of leads (I, II, III, aVR, aVL, aVF – like vs. three leads, respectively). Therefore, they were presented to cardiologists as separate data sets rather than merged into one. Continuous variables were reported as mean with standard deviation or median with interquartile range. Categorical variables were presented as counts and percentages. For diagnostic performance evaluation we applied standard measures such as sensitivity, specificity, accuracy, positive likelihood ratio and negative likelihood ratio. Due to the great dependence on the prevalence of disease, positive or negative predictive values were not evaluated. An independent sample Student's *T*-test or Mann-Whitney U test was applied to quantitative data. When the expected values in any of the cells of a contingency table were ≥5, a Chi-square test was applied for categorical data. Otherwise, a two-tailed Fisher's exact test was selected. Cramer's V was used to measure the association between results of investigated diagnostic methods and reference. Data was processed using the statistical package for the social sciences (27.0, SPSS Inc., Chicago, IL, USA).

## Results

**Figure 3:**
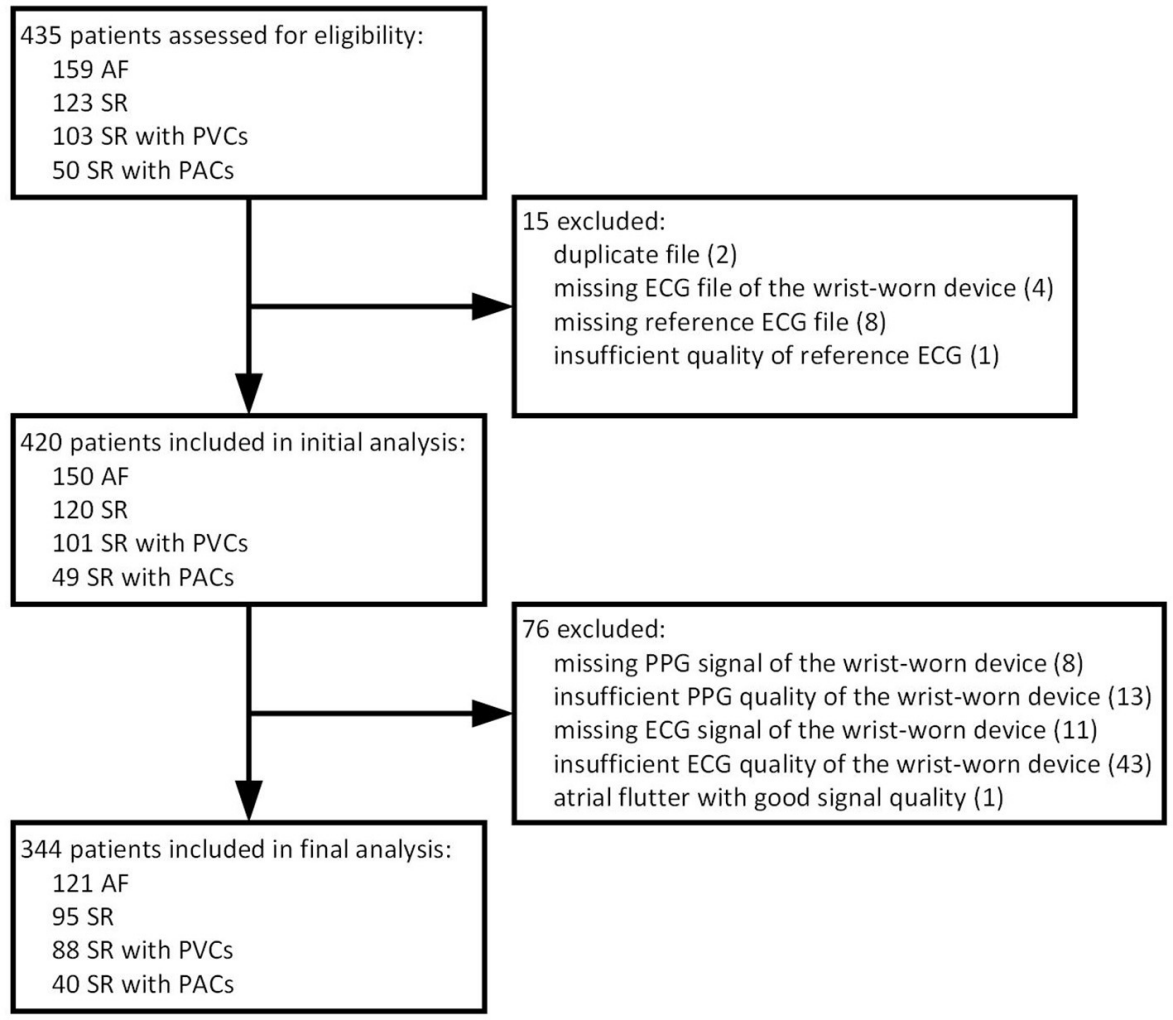
Flow chart of patients. AF, atrial fibrillation; SR, sinus rhythm; PVC, premature ventricular contraction; PAC, premature atrial contraction; ECG, electrocardiography; PPG, photoplethysmography.

**Figure 4:**
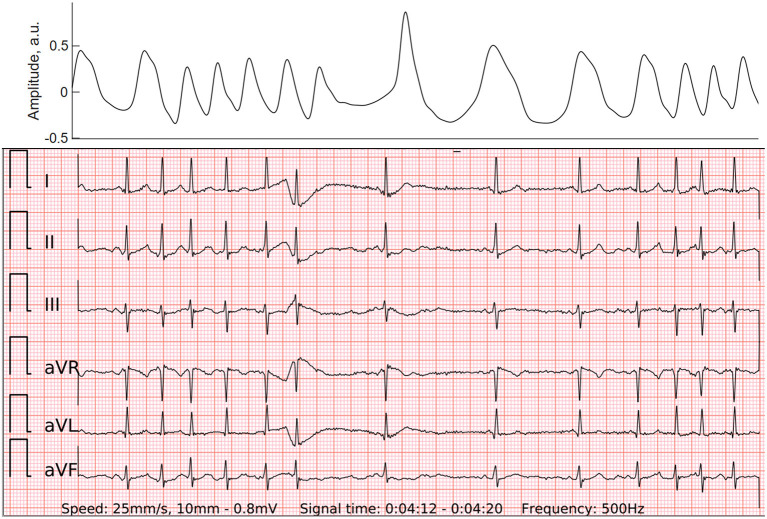
PPG (top panel) and wearable 6-lead ECG (lower panel) of the atrial run, which may also be called “micro-AF”. By definition, it is a sudden onset of irregular tachycardia with episodes of ≥5 consecutive supraventricular beats and total absence of P-waves, lasting less than 30 s (16).

A total of 435 patients were assessed for eligibility in a single center between March 2019 and September 2019. As presented in detail (Figure 3), we excluded 15 subjects due to logistical errors (duplicates or missing data files). After the initial analysis of PPG tracings, 8 patients were excluded due to missing PPG signals and 13 due to insufficient PPG quality. Regarding the wearable ECG method, we excluded 11 subjects due to missing ECG signals and 43 due to insufficient ECG quality. One patient was excluded due to typical atrial flutter instead of AF. Therefore, the final sample size constituted 344 patients. Our analysis included 121 patients with AF, predominantly paroxysmal, 95 patients with stable SR and 128 patients with SR and frequent premature contractions (Table 1). The latter group consisted of individuals with dominant PVCs (*n* = 88) or PACs (*n* = 40). To meet a threshold for a sufficient frequency of at least one extrasystole per 2 min, a Holter ECG of validated device was thoroughly examined. The real burden of PVCs/PACs exceeded the threshold to a large extent and comprised a median of 6.2 (16.1–2.8) premature beats per minute. Importantly, almost a quarter of this group (31/128, 24.2%) had episodes of bigeminy or trigeminy and almost a one-tenth (12/128, 9.4%) had runs of ≥3 PACs/PVCs which were often irregular (Figure 4). The mentioned arrhythmogenicity parameters reflect the significant pressure we have put on both the PPG algorithm and the ECG of the device to differentiate between SR and AF.

**Table 1:** Baseline characteristics.

**Characteristic**	**AF (*n* = 121)**	**Stable SR (*n* = 95)**	**SR with frequent premature contractions (*n* = 128)**
Age (yrs.), mean ± SD	65.6 ± 11.2	64.0 ± 13.8	67.3 ± 14.2
Male, *n* (%)	64 (52.9)	55 (57.9)	69 (53.9)
Paroxysmal: persistent: Permanent AF	101:14:6	NA	NA
Type and frequency of premature contractions			
Dominant PVC: dominant PAC type	NA	NA	88:40
Cases with frequent runs of ≥3 PACs/ PVCs, *n* (%)	0 (0)	0 (0)	12 (9.4)
Cases with frequent bigeminy/ trigeminy episodes, *n* (%)	0 (0)	0 (0)	31 (24.2)
PVCs, median beats/min (IQR)	<0.5	<0.5	6.7 (16.4–2.6)
PACs, median beats/min (IQR)	<0.5	<0.5	5.5 (14.6–2.9)
Total, median beats/min (IQR)	<0.5	<0.5	6.2 (16.1–2.8)
**CHADS**_**2**_ **VASc risk score (categorical)**			
0–1, *n* (%)	37 (30.6)	4 (18.2)^a^	1 (3.2)^b^
2–4, *n* (%)	64 (52.9)	14 (63.6)^a^	21 (67.7)^b^
≥5, *n* (%)	20 (16.5)	4 (18.2)^a^	9 (29)^b^
CHADS_2_VASc risk score (quantitative), mean ± SD	2.7 ± 1.7	3.1 ± 1.4^a^	3.8 ± 1.7^b^
HAS-BLED score, mean ± SD	0.9 ± 0.8	0.8 ± 0.6^a^	1.4 ± 1.0^b^
OAC, *n* (%)	91 (75.2)	19 (20)	23 (18)
DOAC, *n* (%)	67 (55.4)	15 (15.8)	15 (11.7)
Warfarin, *n* (%)	23 (19)	4 (4.2)	8 (6.3)
LMWH, *n* (%)	1 (0.8)	0 (0)	0 (0)

a *Calculated for patients with a history of AF, thus the denominator is 22*.

b *Calculated for patients with a history of AF, thus the denominator is 31*.

*AF, atrial fibrillation; SR, sinus rhythm; PVC, premature ventricular contraction; PAC, premature atrial contraction; OAC, oral anticoagulant; DOAC, direct oral anticoagulant; LMWH, low molecular weight heparin; IQR, interquartile range*.

### Performance of the Automated PPG-Based Algorithm of the Device for AF Detection

**Figure 5:**
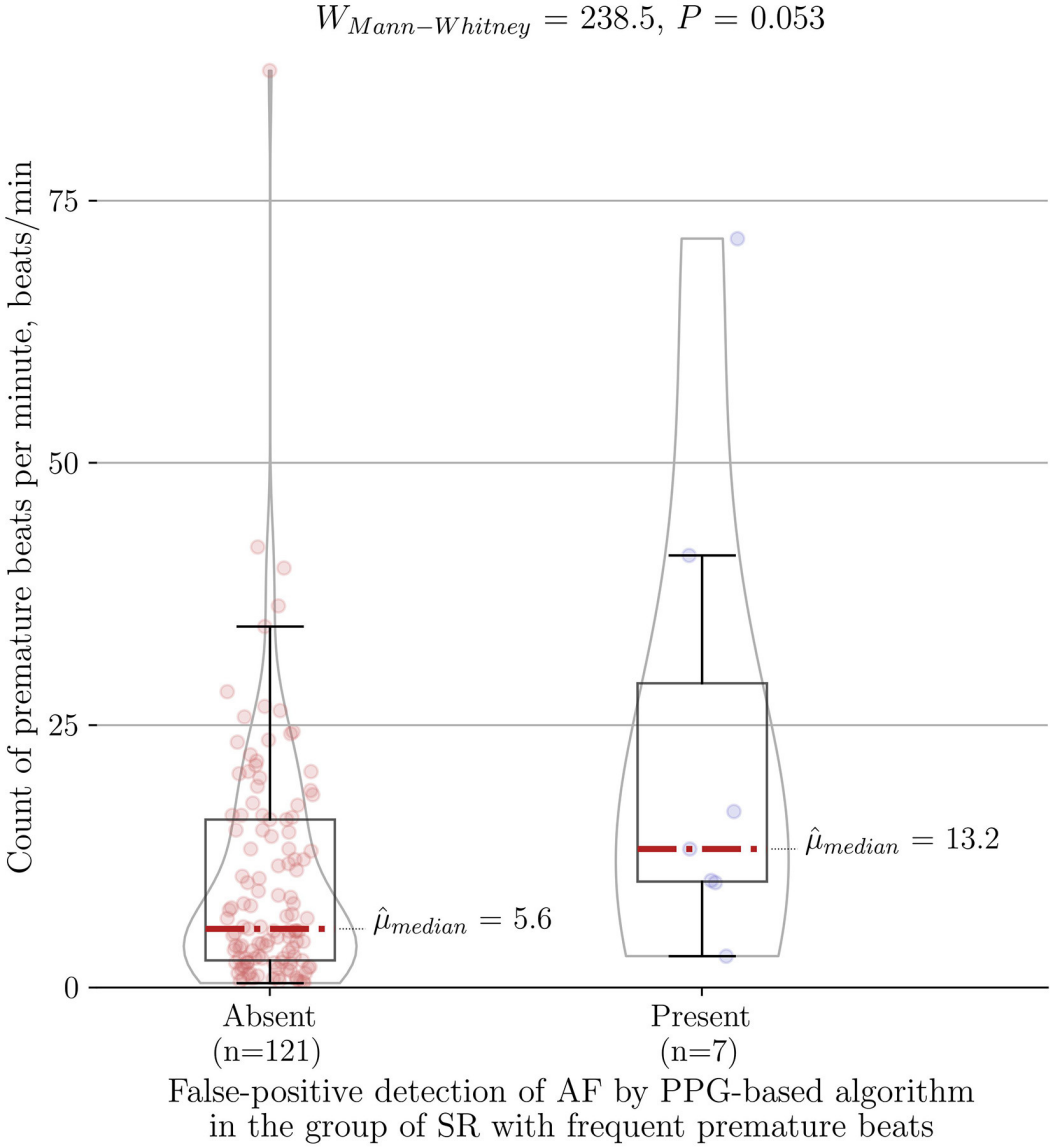
Association between count of premature beats per minute and type I error of the PPG-based algorithm for AF detection in the control group of SR with frequent premature beats (*n* = 128). PPG, photoplethysmography; AF, atrial fibrillation; SR, sinus rhythm.

The total duration of the PPG recordings of 344 individuals was 8933.8 min, averaging 26.0 ± 29.9 min per patient. The PPG-based detector embedded in the wrist-worn device analyzed the rhythm and successfully detected AF with a sensitivity of 94.2%, specificity of 100% and accuracy of 99.9% when AF was compared to the stable SR group (Table 2). In addition, if we included patients with frequent PVCs/PACs into the control group, it resulted in seven false-positive cases (three due to frequent PVCs and four due to frequent PACs) compared to none in stable SR group (*P* < 0.001). Consequently, sensitivity, specificity, and accuracy dropped to 94.2, 96.9, and 96.8%, respectively. As anticipated, the median of premature beats per minute in our false-positive cases of the PPG-based algorithm reached 13.2 (IQR 41.2–10), (*n* = 7) and tended to be higher compared to the burden of ectopy in the rest of cases in the group of SR with frequent PVCs/PACs, which comprised 5.6 (IQR 16–2.5), (*n* = 121) (*P* = 0.053) (Figure 5). In contrast, among false-positive cases subgroup none of the patients had bigeminy/trigeminy episodes (0 of 7, *P* = 0.124) and only a minority had frequent runs of ≥3 PACs/PVCs (2 of 7, *P* = 0.073).

**Table 2:** Diagnostic measures of automated PPG-based algorithm for AF detection.

**Measure**	**AF vs. stable SR group (*n* = 216)**	**AF vs. both SR groups including frequent PVCs/PACs (*n* =344)**
Sensitivity (%), (95% CI)	94.2 (88.4–97.6)	94.2 (88.4–97.6)
Specificity (%), (95% CI)	100 (96.2–100)	96.9 (93.6–98.7)
Accuracy (%), (95% CI)	99.9 (98.2–100)	96.8 (94.4–98.4)
LR (+), (95% CI)	-	30.01 (14.46–62.31)
LR (-), (95% CI)	0.06 (0.03–0.12)	0.06 (0.03–0.12)

*AF, atrial fibrillation; SR, sinus rhythm; PVC, premature ventricular contraction; PAC, premature atrial contraction; LR (+), positive likelihood ratio; LR (-), negative likelihood ratio*.

### Performance of the 6-Lead-ECG of the Device for AF Detection When Assessed by Independent Cardiologists

When three diagnosis-blinded cardiologists assessed wearable ECG recordings (*n* = 344), three of them were classified as “Cannot be concluded” (*n* = 3). The rest of the tracings (*n* = 341) yielded a sensitivity of 99.2%, a specificity of 100% and an accuracy of 100% when comparing the AF group vs. the stable SR group (Table 3). Extending the control group with all SR patients including frequent PVCs/PACs led to a sensitivity of 99.2, a specificity of 99.1, and an accuracy of 99.1%. Similarly to PPG-based AF detection results, the group of SR with frequent premature contractions here added two false-positive cases and thus slightly decreased the specificity. However, the difference of type I error due to inclusion of patients with frequent PVCs/PACs was not significant compared to the stable SR group (*P* = 0.065).

**Table 3:** Diagnostic measures of the 6-lead ECG of the device for the detection of AF.

**Measure**	**AF vs. stable SR group (*n* = 214)**	**AF vs. both SR groups including frequent PVCs/PACs (*n* = 341)**
Sensitivity (%), (95% CI)	99.2 (95.4–100)	99.2 (95.4–100)
Specificity (%), (95% CI)	100 (96.2–100)	99.1 (96.8–99.9)
Accuracy (%), (95% CI)	100 (-)	99.1 (97.4–99.8)
LR (+), (95% CI)	-	110.07 (27.70–437.41)
LR (-), (95% CI)	0.01 (0.00–0.06)	0.01 (0.00–0.06)

*AF, atrial fibrillation; SR, sinus rhythm; PVC, premature ventricular contraction; PAC, premature atrial contraction; LR (+), positive likelihood ratio; LR (-), negative likelihood ratio*.

### Performance of the Integrated System of the PPG-Based Algorithm and the 6-Lead-ECG of the Device for AF Detection

**Figure 6:**
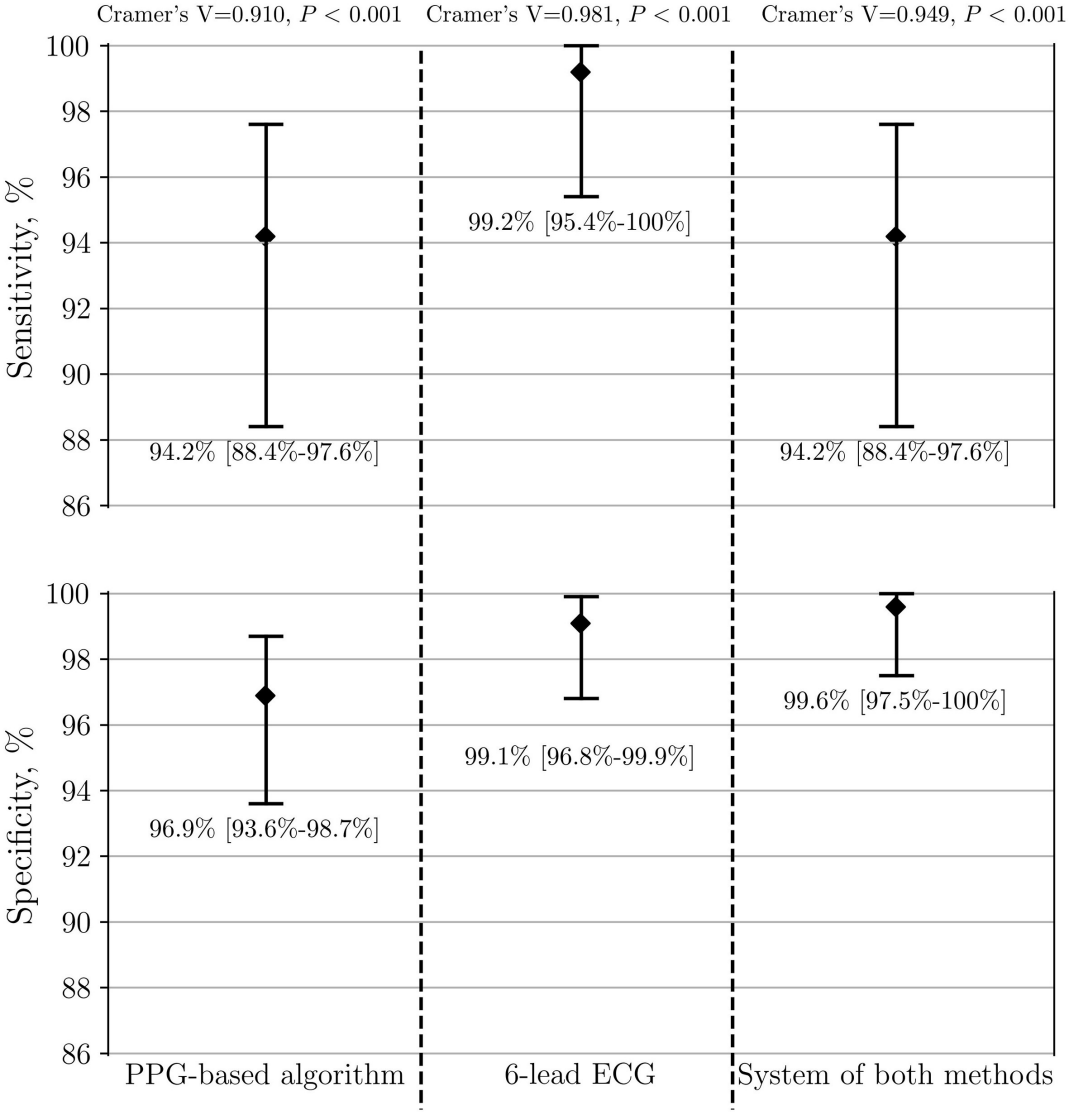
Performance of PPG-based algorithm, 6-lead ECG and the system of both methods to detect AF (*n* = 341). The group of AF is compared to both control SR groups, including patients with frequent PVCs/PACs. PPG, photoplethysmography; ECG, electrocardiography; AF, atrial fibrillation; SR, sinus rhythm; PVC, premature ventricular contraction; PAC, premature atrial contraction.

The model of integrated “double-check” system with both methods together, as described in the measurements section, yielded a sensitivity of 94.2, a specificity of 100, and an accuracy of 99.9% when differentiating between AF vs. stable SR (Table 4). Furthermore, comparing AF vs. all patients with SR including frequent PVCs/PACs led to a sensitivity of 94.2, a specificity of 99.6% and an accuracy of 99.5%. Of seven initially false-positive cases by the PPG-based algorithm, the diagnosis-blinded cardiologists were able to correct the diagnosis to SR in six of them (*P* = 0.012). The system of both methods demonstrated a high Cramer's V association (0.949, *P* < 0.001) (Figure 6).

**Table 4:** Diagnostic measures of the system combining monitoring with an automated PPG-based algorithm together with the 6-lead wearable ECG confirmation.

**Measure**	**AF vs. stable SR group (*n* = 216)**	**AF vs. both SR groups including frequent PVCs/PACs (*n* 344)**
Sensitivity (%), (95% CI)	94.2 (88.4–97.6)	94.2 (88.4–97.6)
Specificity (%), (95% CI)	100 (96.2–100)	99.6 (97.5–100)
Accuracy (%), (95% CI)	99.9 (98.2–100)	99.5 (98.0–100)
LR (+), (95% CI)	-	210.10 (29.71–1485.76)
LR (-), (95% CI)	0.06 (0.03–0.12)	0.06 (0.03–0.12)

*AF, atrial fibrillation; SR, sinus rhythm; PVC, premature ventricular contraction; PAC, premature atrial contraction; LR (+), positive likelihood ratio; LR (-), negative likelihood ratio*.

## Discussion

To our knowledge, this is the first study of a wearable that offers a combination of continuous PPG for screening of AF together with the possibility of recording an intermittent 6-lead standard-limb-like ECG without any wires for rhythm confirmation by a physician. The authors evaluated the performance of the device with particular emphasis on the challenge of ectopic contractions that is often underestimated. Frequent premature beats reduced the specificity of the PPG algorithm and should be routinely considered when realistically evaluating emerging mHealth technologies. Furthermore, the 6-lead ECG with high sensitivity and specificity despite frequent ectopic contractions added great value to reduce type I errors of the PPG-based algorithm and integrate into one system of both methods.

### Impact of Premature Contractions on the PPG-Based Algorithm for AF Detection

The PPG-based AF detection algorithms are increasingly used in wearables and apps. However, there is a lack of device validation studies that comprehensively test the algorithms on patients with frequent premature contractions. Choosing a stable SR control group underrepresents real-world settings. Subsequently, some physicians express a low trust in the specificity of wearables in daily practice. It may contribute to denying reimbursement of wearable diagnostics, reported in a recent survey by as much as 36% (194/539) of respondents worldwide (14). Therefore, we dedicated a distinct control group and reported a comprehensive analysis of premature contractions as beats per minute, cases with frequent runs of PACs/PVCs or bigeminy/trigeminy episodes. To compare different technologies and acquire reproducible results such standard of reporting is highly needed. One of our key findings is that frequent PVCs/PACs reduce the specificity of the PPG-based AF detection algorithm as all seven false-positive cases were from this group in contrast to none from the stable SR group. The authors further investigated what qualitative and quantitative characteristics of premature beats were associated with type I error of the PPG-based algorithm. Interestingly, cases with bigeminy/trigeminy episodes as well as frequent runs of ≥3 PACs/PVCs did not reduce the diagnostic accuracy of the algorithm. Such finding could be explained by a dedicated bigeminy suppression in the algorithm. However, a type I error of the PPG-based algorithm had a tendency to be associated with a higher burden of premature beats per minute (Figure 5).

Frequent premature contractions seem to be a specificity lowering factor for Apple Watch PPG-based notification for irregular rhythm detection. A recent substudy of participants with an irregular pulse notification on the Apple Watch and no AF observed on ECG patch revealed other atrial or ventricular arrhythmias (mostly ectopic beats) in 40% of participants (15). This is important to acknowledge when applying similar PPG-based apps or devices for wide population research such as Apple Heart Study (5) or TeleCheck-AF project (4). Even if it is considered a screening measure it may potentially cause harmful effects for an individual with many PVCs/PACs, e.g., unnecessary visits, interventions, anxiety. The authors are strongly convinced that in the field of wearables with a continuous PPG algorithm for the screening of AF, specificity is of critical importance. Since an individual is unobtrusively monitored for a prolonged period of time, there is a high chance of arrhythmia detection and, thus, novel technologies should be in line with the principle of “first, do no harm.”

### Impact of Premature Contractions on the ECG of the Wearable for AF Detection

The effect of premature beats on the ECG-based AF detection was minor in our study. The 6-lead ECG of the wearable significantly reduced type I errors of the PPG-based algorithm. Both false-positive wearable ECG cases were particular tracings of SR with multiple and irregular runs of PACs. These episodes may arguably represent the initial stage of the development of AF or an undiagnosed conventional AF. The STROKESTOP study group from Karolinska Institute coined the term 'micro-AF' for this phenomenon. By definition, it is a sudden onset of irregular tachycardia with episodes of ≥5 consecutive supraventricular beats and total absence of P-waves, lasting less than 30 s (16). In a large-scale AF screening study of 7,173 individuals micro-AF was related to a higher risk of AF (HR 4.3; 95% CI 2.7–6.8) and death (HR 2.0; 95% CI 1.1–3.8) (17). Furthermore, a single false-negative case in our study presented with f-waves which occasionally organized and imitated regularly irregular P-waves. Hence it gave the diagnosis-blinded cardiologists a hard time to differentiate it from SR with PACs. Interestingly, short strips of reference Holter ECG corresponding to these two false-positive and one false-negative cases were also falsely recognized as AF and SR, respectively. Only after thorough examination of long reference Holter ECG tracings cardiologists were able to confirm the diagnosis of these challenging cases. It suggests that diagnostic measures of a 6-lead-ECG of the wearable without any wires could be non-inferior to regular 3-lead Holter ECG recordings.

### Impact of Premature Contractions on Automated ECG Algorithm for AF Detection

The impact of frequent PVCs/PACs on the accuracy of automated ECG algorithms in available devices is likewise important. Although implantable loop recorders are considered a reliable tool for prolonged monitoring with great compliance, their algorithm for arrhythmia detection from a single-lead ECG has been reported to produce substantial numbers of false-positive results. In a prospective study of 559 participants, the incidence of false-positive transmissions was as high as 46% (201/440) for patients with AF surveillance indication for implantable loop recorder (18). Among the different categories of false-positive cases in scheduled and alert transmissions the proportion of falsely diagnosed AF was 50 and 32%, respectively. The paramount etiology of false-positive cases in alert transmissions was premature ventricular or atrial ectopy (52%). The average workload to review one false-positive transmission and make a decision after consulting electrophysiologists was estimated between 30 and 45 min.

### Impact of Premature Contractions on PPG-Based Algorithm vs. ECG for AF Detection

Although the 6-lead ECG of the wearable in our study tended to perform better compared to the automatic PPG algorithm (sensitivity 99.2 vs. 94.2%, specificity 99.1 vs. 96.9%), the difference was not statistically significant as the confidence intervals overlapped. Similarly, Gruwez et al. (19) compared detection of AF from a single-lead ECG vs. PPG waveform after manual interpretation by physicians. Despite the small number of participants (*n* = 30) it was a commendably rare example of a distinguished SR group with extrasystoles. The lone PPG waveform yielded a rather small sensitivity of 88.8% and a specificity of 86.3%. Only after adding tachogram and Poincaré plot the PPG-based detection increased the sensitivity to 95.5% (*P* < 0.001) and the specificity to 92.5% (*P* < 0.001). Then it did not show any significant difference from the sensitivity and the specificity of single-lead ECG manual interpretation for the detection of AF, 91.2% (*P* = 0.67) and 93.9% (*P* = 0.54), respectively. Whether the equivalent outcome would occur after comparing the manual PPG interpretation with the 6-lead ECG of our wearable remains to be investigated. Although the PPG algorithm appears to be a suitable method for screening, current AF guidelines remain restricted to only a standard 12-lead ECG or ECG strip with AF of at least 30 s (including wearable-recorded ECGs) to establish the diagnosis by the physician (2). In our study both methods are predominantly assigned to these two different purposes, i.e., the PPG-based algorithm is assigned for screening of AF and the 6-lead ECG is assigned for rhythm confirmation. Therefore, both methods work synergistically in the wrist-worn device.

### Limitations

**Figure 7:**
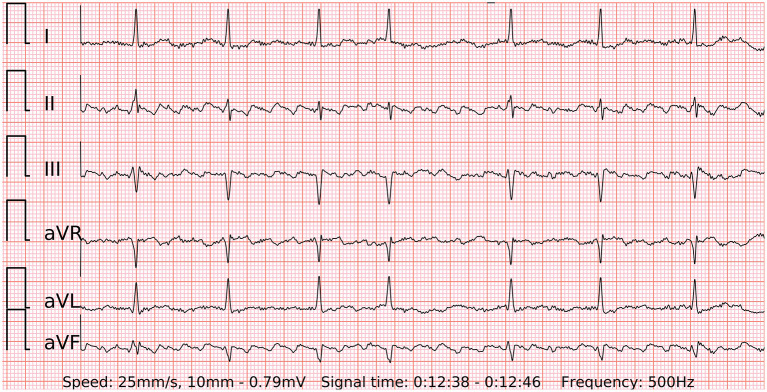
A 6-lead ECG of typical counterclockwise atrial flutter with variable atrioventricular conduction recorded by the wearable device.

The generalizability of this study might be limited to the involved population. As outlined in a comprehensive review (20), the accuracy and cost-effectiveness of mHealth technologies for AF detection depend greatly on given incidence, risk profile, type of AF and other characteristics. For instance, all the participants in the presented study were White. Diverse skin pigmentation may alter the results of PPG-based AF detection. Predefined groups of patients with AF and SR may hypothetically produce bias. Patients with atrial flutter were beyond the scope of this study and may present with different patterns of the pulse wave, though the wearable 6-lead ECG seems to be a promising diagnostic tool for future investigations in such subjects (Figure 7). A part of recordings was not analyzed due to presented reasons (Figure 3) and may cause additional costs or visits for the patients in real-life conditions. Furthermore, the results were derived from an analysis of short-term in-hospital recordings. Diagnostic measures of the device in outpatient settings may differ. In particular, the quality of PPG has been reported to decrease significantly during most daily activities but has a reasonably good quality for analysis during sleep (21). Therefore, it can be anticipated that the accuracy of AF screening should be higher during the sleep state.

## Conclusions

This is a validation study of the prototype of the wearable device that offers a combination of a PPG-based AF detection algorithm for the screening of AF and an instant 6-lead ECG without any wires for rhythm confirmation. The system maintained high specificity for AF detection despite a remarkable amount of frequent single or multiple premature contractions in the control group. The ability of the device to accurately detect AF in long-term screening and allow the physician to confidently diagnose the arrhythmia without further testing remains to be investigated.

## Data Availability Statement

The raw data supporting the conclusions of this article will be made available by the authors, without undue reservation.

## Ethics Statement

The studies involving human participants were reviewed and approved by Vilnius regional bioethics committee with registration No. 158200-18/7-1052-557. The patients/participants provided their written informed consent to participate in this study.

## Author Contributions

JB, AA, and VM were responsible for design and general execution of the study. JB, ZA, ED, DA, MP, JM, JS, and NB were involved in search and inclusion of patients. AK, VJ, RJ, and AS analyzed the recorded data. AS, DS, AP, MB, BP, SD, and AR implemented the algorithm and technological solutions of the device. EJ was involved in statistical analysis. JB wrote the initial manuscript. All authors reviewed and edited the manuscript.

## Funding

This work was supported by the European Regional Development Fund under agreement with the Research Council of Lithuania (01.2.2-LMT-K-718-03-0027).

## Conflict of Interest

JB, AS, DS, AP, MB, SD, VM, and AA hold patent for the technology. JB received travel grants from Abbot, consults Teltonika Telemedic, received travel grants from Biosense Webster. The remaining authors declare that the research was conducted in the absence of any commercial or financial relationships that could be construed as a potential conflict of interest.

## Publisher's Note

All claims expressed in this article are solely those of the authors and do not necessarily represent those of their affiliated organizations, or those of the publisher, the editors and the reviewers. Any product that may be evaluated in this article, or claim that may be made by its manufacturer, is not guaranteed or endorsed by the publisher.
